# Retrospective evaluation of notched and fragmented QRS complex in dogs with naturally occurring myxomatous mitral valve disease

**DOI:** 10.1080/01652176.2021.1992803

**Published:** 2021-10-25

**Authors:** Radu Andrei Baisan, Cătălina Andreea Turcu, Eusebiu Ionuț Condurachi, Vasile Vulpe

**Affiliations:** Clinics Department, Faculty of Veterinary Medicine, University of Applied Life Sciences “Ion Ionescu de la Brad”, Iași, Romania

**Keywords:** Canine, dog, myxomatous mitral valve disease, electrocardiogram, QRS complex

## Abstract

Myxomatous mitral valve disease (MMVD) is the most common cardiac disease in dogs. The association of QRS notching (nQRS) or fragmentation (fQRS) with disease severity is currently unknown. The study objective was to assess the prevalence of nQRS and fQRS in dogs with MMVD and its severity according to ACVIM classification and to compare the results with a group of healthy dogs. This retrospective cross-sectional study included 34 healthy control dogs and 155 dogs with spontaneous MMVD (42% of dogs in class B1, 23% in class B2 and 35% in class C). fQRS was defined as nQRS complexes in two contiguous leads in the frontal plane (leads I and aVL) and (II, III or aVF). A one-way ANOVA with Bonferroni post-hoc test was used to assess the differences in continuous data between control and MMVD groups. Of the MMVD group, 58% showed nQRS in at least one lead and 27% presented fQRS. There was no difference between the number of leads with a nQRS and disease severity (*p* = 0.75) nor did the number of leads with a nQRS correlate with left atrial size (*r* = 0.48; *p* = 0.5). The number of dogs with fQRS did not differ among classes of MMVD (*p* = 0.21). nQRS and fQRS were more prevalent in dogs with MMVD compared to control dogs (*p* < 0.01). This study did not identify any relationship between the number of leads with a nQRS and disease severity. However, dogs with MMVD had a higher prevalence of nQRS and fQRS compared to control group.

## 1. Introduction

Myxomatous mitral valve disease (MMVD) is the most frequently acquired cardiac disease in dogs (Keene et al. 2019) characterized by progressive degeneration of the mitral valve resulting in mitral regurgitation and cardiac volume overload (Sakarin et al. 2016). One study had shown that out of 85 dogs with a notched QRS complex, 30 were affected by MMVD. However, the association of these changes with the disease severity has not been investigated yet (Winter and Bates 2018). In human medicine, notching of the QRS complex has been studied in various cardiovascular diseases being associated with worse prognosis (Morita et al. 2008; Yuce et al. 2010; Bekar et al. 2019; Bi et al. 2020; Canpolat et al. 2020; Tekin Tak et al. 2020; Yooprasert et al. 2020). It was suggested that the alteration in QRS morphology is related to regional myocardial scar, leading to a conduction delay or a fragmentation of QRS complexes (Flowers et al. 1969; el-Sherif 1970). Fragmented QRS (fQRS) however, associated with myocardial fibrosis is described in humans as notching of the QRS complex in at least two contiguous leads (V1–V5 for the anterior segment, I, aVL, and V6 for the lateral segment, or II, III, and aVF for the inferior segment), corresponding to the left ventricular (LV) wall (Das et al. 2010; Jain et al. 2014). Similar to humans, in dogs, lead orientation in the frontal plane relative to the vectors orientation explains the morphology of the recorded waves in each lead. Leads II, III and aVF examine the cardiac activation wave-front from an inferior position, while leads I and aVL record the electrical activation from a left lateral position (Santilli and Perego 2014).

In veterinary medicine, QRS complex notch (nQRS) has been described in several cardiac conditions such as interventricular septal defect, tricuspid valve dysplasia, pulmonic stenosis, arrhythmogenic right ventricular cardiomyopathy, left ventricular concentric hypertrophy, cardiac neoplasia, as well as in myocarditis secondary to viral infection or canine babesiosis (Robinson et al. 1979; Armstrong et al. 1986; Kornreich and Moise 1997; Dvir et al. 2004; Scurtu et al. 2017; Winter and Bates 2018). Infiltrative conditions such as cardiac neoplasia, dilated cardiomyopathy or myocarditis are associated with necrosis, inflammatory cell infiltrate, hemorrhage and fibrin microthrombi which may induce a conduction delay within the ventricles (Dvir et al. 2004; Supreeth and Francis 2020). However, MMVD in dogs is mainly associated with cardiac remodeling due to volume overload as a consequence of mitral regurgitation and increased left atrial pressure (Keene et al. 2019). Thus, the association and prevalence of QRS abnormalities in this pathology is currently unknown. We hypothesize that notched and fragmented QRS complex would not be associated with the disease severity in dogs with naturally occurring MMVD since this condition does not comprise a primary myocardial dysfunction associated with wide fibrotic areas.

Therefore, the aim of the study is to assess the prevalence of nQRS and fQRS in dogs with MMVD and its severity according to ACVIM classification and to compare the results with a group of healthy dogs.

## 2. Materials and methods

Data generated between 2017 and 2020 were retrospectively collected from the Cardiology Service database. This retrospective cross-sectional study included client-owned dogs with naturally occurring MMVD divided into three (B1, B2 and C) groups, according to the ACVIM classification (Keene et al. 2019). Dogs were allocated in stage B1 if there was an audible left apical systolic murmur along with evidence of mitral valve thickening and presence of a mitral regurgitating jet in the absence of cardiomegaly defined as a La/Ao > 1.6 and LVIDd_n > 1.7. Dogs were allocated in stage B2 if there was evidence of left sided cardiomegaly defined as a La/Ao > 1.6 and LVIDd_n > 1.7 and a VHS score above 10.5 associated with signs of mitral valve insufficiency in the absence of evidence of radiographic pulmonary edema, while dogs in stage C were allocated if there was evidence of left sided cardiomegaly along with presence of tachypnea and evidence of pulmonary edema on radiography. All dogs in stage C included in this study were at their first onset of CHF therefore considered acute stage C.

A group of healthy dogs (N), referred for cardiologic examination prior to anesthesia for different non-cardiac related interventions, selected in agreement to the study group regarding age and sex, was used as control. Dogs in control group were considered healthy based on physical examination (normal heart and respiratory rate, presence of sinus arrhythmia and absence of any cardiac murmurs), a normal electrocardiographic tracing and echocardiographic study (normal aspect of the mitral valve leaflets, absence of a regurgitating jet and absence of left sided cardiomegaly) and thoracic radiography without signs of cardiac enlargement or pulmonary edema. Dogs with metabolic or systemic diseases suspected based on physical examination were excluded.

In the study group, only dogs below 25 kg with a left apical systolic murmur, echocardiographic evidence of MMVD, and Doppler evidence of a mitral regurgitation jet, were respectively included. Only dogs with a good quality electrocardiographic trace recording in six limb leads (I, II, III, aVR, aVL and aVF) were accepted for analysis. Patients with sustained arrhythmia, wide QRS complexes (>70 msec) and artifacts that could influence the evaluation of the QRS complex were excluded. When multiple electrocardiograms (ECGs) were recorded from the same patient, only the one from the first presentation was included for analysis.

All dogs received a complete physical examination, a five-minute-long, computerized ECG recording (PolySpectrum 8E/8V, Neurosoft, Ivanovo, Russia) using six standard limb leads, thoracic radiography, and a complete echocardiographic study (Logiq V5, GE Medical Systems, Wuxi, China) using a 4-7 MHz phased-array transducer.

**Figure 1.**
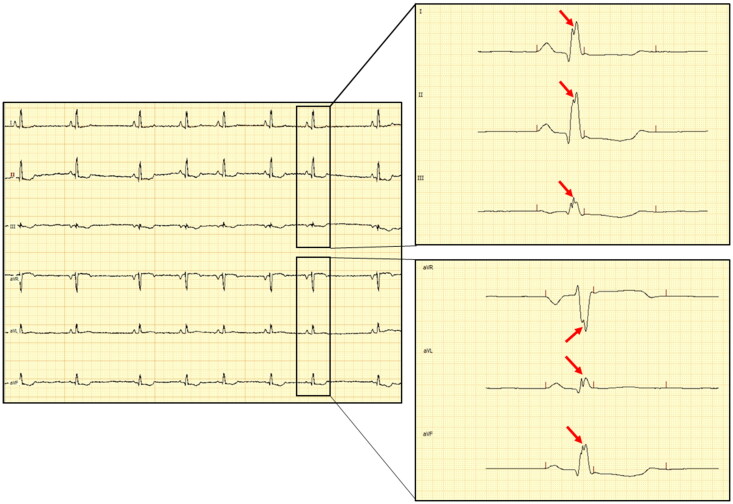
Electrocardiographic tracing of a dog with fQRS: left – figure of the 6-lead electrocardiographic tracing calibrated at 10 mm/mV and 50 mm/sec; right – selected captions during interpretation of fragmented QRS calibrated at 200 mm/sec and 20 mm/mV – note the slurring in the peak of the R-wave characterized as a double-peaked R-wave (red arrows) in all six limb leads (leads I, II and III in the upper-right corner and aVR, aVL and aVF in the lower-right corner).

Dogs underwent ECG recordings in right lateral recumbency following accommodation to a quiet room, and prior to the echocardiographic examination. Electrodes were placed on the limbs (Tilley et al. 2016) and the recording was performed at 50 mm/s and 10 mm/mV for a five-minute period as an in-house protocol. Electrocardiographic analyses were performed with a proprietary, dedicated software (PolySpectrum-Neurosoft v.4.8.118) using an electronic caliper. The QRS complex analysis was performed by a single operator (BRA) in all six leads at 200 mm/s and 20 mm/mV to ensure a proper visual resolution. Notched QRS was defined as (1) an additional R wave (R prime), (2) notching in nadir of the S wave, (3) notching of R wave, or (4) the presence of more than 1 R prime in at least one limb lead (Bi et al. 2020) while the fQRS was defined as nQRS complexes in two contiguous leads in the frontal plane (leads I and aVL) corresponding to the left-anterior area of the left ventricular wall (VLAA) and (II, III or aVF) corresponding to the posterior area of the left ventricular wall (VPA) (Santilli and Perego 2014). An example of ECG tracing with fragmented QRS complex is presented in Figure 1.

Echocardiography was performed as previously described (Thomas et al. 1993) by a single investigator, with nine years of experience in veterinary cardiology working as referral (BRA) and included subjective evaluation of valve structure and function, color Doppler interrogation of the mitral regurgitation jet at the left parasternal apical four-chamber view, right parasternal short axis view of the left atrial dimension scaled to the peak diastolic aortic root diameter (LA/Ao), left ventricular diameters in systole (LVIDs-n) and diastole (LVIDd-n) indexed to the body-weight (BW) (Cornell et al. 2004) and shortening fraction (SF%). Cardiomegaly was identified based on the following criteria: LA/Ao > 1.6, LVIDd-n > 1.7 and a radiographic vertebral heart scale (VHS) value above the breed reference range or > 10.5 where breed-specific limits were unavailable (Keene et al. 2019). Thoracic radiography along with the respiratory rate on time of presentation were used to establish the presence of pulmonary edema (Rishniw et al. 2012).

For the study group, the sample size was calculated according to literature to 160 dogs assuming the size of the expected effect (r_m_) of 0.25 with a 90% level of statistical power at an alpha level of 0.05 (Friedman 1982). In the same manner, for the control dogs, assuming an expected size effect (r_m_) of 0.45 with an 80% level of statistical power at an alpha level of 0.05 the minimum sample size was calculated at 33 dogs.

Statistical analysis was performed using SPSS v.17 (IBM USA) and GraphPad Prism v.5 (GraphPad, San Diego, CA, USA). Data were tested for normality using the Shapiro-Wilk test. Normally distributed data are expressed as mean ± SD. A one-way ANOVA with Bonferroni post-hoc test was used to assess the differences in continuous data (age, body-weight) in control and MMVD groups. Analyses of relative proportions of sex, and proportion of nQRS leads and fQRS between control group and groups divided by ACVIM classes were performed using the Pearson Chi-Square test and the Fisher’s exact test. Correlations between the LA/Ao ratio (used as a surrogate for MMVD severity beside ACVIM classification) and number of notched QRS leads were assessed using the Spearman’s rank correlation coefficient (r). Results were considered significant when P < 0.05.

Ethical approval was obtained from the Ethics Committee of the Faculty of Veterinary Medicine of Iași (number 28/2021, 18^th^ of January 2021).

## 3. Results

A total of 189 dogs were selected based on inclusion criteria. The control group included thirty-four dogs and the study group included 155 dogs divided according to ACVIM classification as following: 65 dogs in class B1, 36 dogs in class B2 and 54 in class C. Age, sex and body-weight characteristics on the entire population and groups divided by ACVIM classification are presented in Table 1. There was no difference in age and gender between groups, however dogs in stage B2 and C had a lower mean body-weight compared to dogs in control group (P < 0.05). There was no difference in age (p = 0.25) and sex (p = 0.2) among groups of dogs with MMVD divided according to ACVIM classes. Dogs belonged to the following breeds: Maltese (n = 62), mix breed (n = 37), Cavalier King Charles Spaniel (n = 29), Poodle (n = 19), Yorkshire terrier (n = 10), Dachshund (n = 8), Pinscher and Chihuahua (n = 6), Spitz, Cocker spaniel and West Highland White Terrier (n = 2), Shih-Tzu, Scottish terrier, Fox terrier, Pug, Chow Chow and Beagle (n = 1).

**Table 1.** Characteristics of the total study population and groups divided by ACVIM classes.

	Total (n = 189)	N (n = 34)	B1 (n = 65)	B2 (n = 36)	C (n = 54)
Age (years)	11.6 ± 2.8	11 ± 2.8	11.5 ± 2.8	11.6 ± 2.8	12.3 ± 2.6
Gender (M/F)	103/86	22/12	29/36	23/13	29/25
BW (kg)	8.45 ± 4.6	8 ± 4.6	8.7 ± 4.9	7.4 ± 2.6^a^	7.4 ± 4.9^b^

M – male, F – female; BW – body-weight.

^a^ Statistical difference between N and B2.

^b^ Statistical difference between N and C.

**Figure 2.**
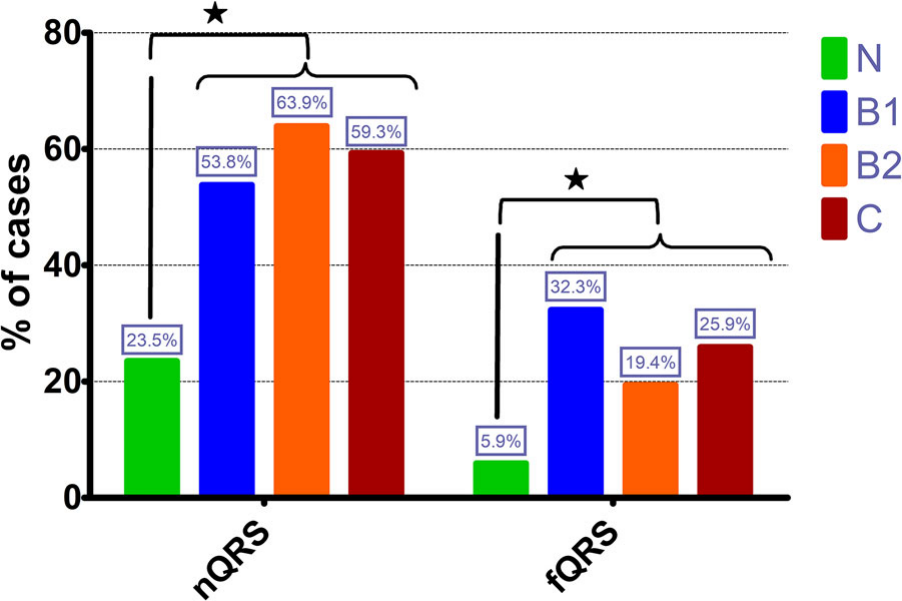
Bar-graphic representing the percentage of healthy dogs and dogs diagnosed with MMVD in different ACVIM classes according to the prevalence of notched QRS (nQRS) and fragmented QRS (fQRS); * – statistically significant difference between control group (N) and all groups of dogs diagnosed with MMVD.

In the control group, 8 dogs (24%) had at least one lead with a notched QRS complex. Of the total group of dogs diagnosed with MMVD, 90 dogs (58%) showed nQRS in at least one lead. The percentage of dogs with a nQRS in control group and with MMVD groups based on ACVIM classification is depicted in Figure 2. The presence of a nQRS was less frequent in control dogs compared to dogs diagnosed with MMVD (p < 0.01).

A nQRS was most frequently present in lead aVL, followed by lead III. The least frequent lead with a nQRS was aVR. The number and percent of dogs on the entire population, control group, and groups divided by ACVIM classes with a nQRS in specific leads are presented in Table 2. There was no significant difference between the number of leads with a nQRS and disease severity based on ACVIM Class (p = 0.75) nor did the number of leads with a nQRS correlate with the left atrial size measured by the La/Ao ratio (r = 0.48; p = 0.5) or with the LVIDd-n (r=-0.38, p = 0.6).

**Table 2.** The number of dogs on the entire population and divided by ACVIM classes with a nQRS in specific leads.

Lead	Total (n/%)	N (n/%)	Class B1 (n/%)	Class B2 (n/%)	Class C (n/%)
Lead I	25/13.2%	0/0%	14/21.5	7/19.4%	4/7.4%
Lead II	18/9.5%	2/5.9%	8/12.3%	2/5.6%	6 /11.1%
Lead III	56/29.6%	2/5.9%	25/38.5%	12/33.3%	17/31.5%
Lead aVL	72/38.1%	5/14.7%	27/41.5%	17/47.2%	23/42.6%
Lead aVR	10/5.3%	2/5.9%	3/4.6%	3/8.3%	2/3.7%
Lead aVF	26/13.8%	2/5.9%	11/16.9%	2/5.6%	11/20.4%

Of the total population, 44 dogs (23%) presented fQRS. In the control group, only two dogs (5.9%) presented fQRS (both dogs with fQRS in VPA). When dogs diagnosed with MMVD were analyzed, forty-two dogs (27%) presented fQRS. Fragmented QRS in VLAA was present in 24 dogs (14 in B1, 6 in B2 and 4 in C) and a fQRS in VPA was present in 25 dogs (10 in B1, 3 in B2 and 12 in C). Out of these dogs, seven had fQRS in both areas (VLAA and VPA) (three in B1, two in B2 and two in C). Control group had a significantly lower number of dogs with fQRS compared to study group (p < 0.01). The percentages of fQRS in control dogs and dogs diagnosed with different MMVD classes, are presented in Figure 2. The number of dogs with fQRS areas (left-anterior or posterior) did not differ among groups based on ACVIM classification (p = 0.21).

## 4. Discussion

The present study aimed to evaluate the frequency of nQRS complexes and fQRS observed on ECG in dogs with naturally occurring MMVD and to assess the association between these electrocardiographic changes and disease severity. In human medicine, the presence of fQRS was associated with various cardiac conditions such as myocardial dysfunction, ischemic and non-ischemic cardiomyopathy, pulmonary hypertension, mitral valve stenosis, atrial septal defect, hypertrophic obstructive cardiomyopathy or Brugada syndrome (Heller et al. 1996; Morita et al. 2008; Das et al. 2010; Yuce et al. 2010; Bekar et al. 2019; Bi et al. 2020), therefore the presence of fQRS in ECG is accepted in human medicine as a marker of myocardial scar or fibrosis (Das et al. 2010).

In the present study, both nQRS and fQRS had a lower frequency in control group when compared to the entire group of MMVD dogs and dogs divided by classes of severity according to ACVIM classification (Keene et al. 2019). One study assessing the myocardial fibrosis by histopathology and Galectin-3 concentration found that the degree of fibrosis in dogs with MMVD was higher than in control group (Sakarin et al. 2016). Another study had shown that dogs with naturally occurring MMVD had significantly more arterial changes in the myocardium, lung, and kidney, and significantly more fibrosis in the myocardium than control dogs. However, the comparison was performed between dogs with CHF and a control group of non-cardiac dogs (Falk et al. 2006). Terho et al. (2014) found that fQRS was present in 20% humans of a large population where 80% of the subjects were healthy. In our study, 23% of the dogs in control group exhibited nQRS on the ECG tracing in at least one lead and 5.9% (2 dogs) exhibited fQRS. Similarly, one previous study in dogs found that out of 85 dogs with a notched QRS in at least one lead, 26 were free of cardiac diseases (Winter and Bates 2018). Both studies raised the hypothesis that in these subjects an early cardiac condition might have been present. In our study, control dogs were subjected to a complete cardiologic examination. As a consequence, this hypothesis is unlikely. Our results suggest that nQRS is commonly present in healthy dogs with an age above 11 years old and body weight below 25 kg.

When dogs with MMVD were analyzed, our study did not show any association between the number of leads with a nQRS and disease severity nor a correlation with the atrial or ventricular size. Furthermore, when contiguous notched QRS leads (fQRS) were analyzed we found a similar prevalence in both (VLAA and VPA). However, there was no correlation between fQRS and disease severity.

To date, only one study evaluated the association between number of leads that presented nQRS and disease severity in dogs with various cardiac conditions, including MMVD, showing that the increasing number of leads with a QRS notch is associated with a higher risk for a cardiovascular dysfunction (Winter and Bates 2018). However, in this study dogs with MMVD were not classified according to disease severity but evaluated as a single group.

Falk et al. (2010) reported a significant correlation between PISA and fibrosis of the papillary muscles in dogs with MMVD suggesting that fibrosis in the papillary muscles may contribute to mitral regurgitation in dogs with MMVD. In our study, all classes of dogs with MMVD had a similar prevalence of nQRS, while fQRS showed mildly higher prevalence in B1 compared to B2 and C, yet none of these electrocardiographic changes did correlate with surrogates for left cardiac enlargement such as LA/Ao ratio or left ventricular internal diameter in diastole.

The results of this study may be explained by the differences in pathological mechanism between MMVD and other conditions associated with myocardial fibrosis. In humans with ischemic heart disease the fibrotic territory is sufficient to generate a terminal conduction delay or a fragmentation of QRS complexes on the 12-lead ECG (Das et al. 2006). Also, fragmented QRS had been associated with hypertrophic cardiomyopathy (Bi et al. 2020), non-ischemic dilated cardiomyopathy (Sinha et al. 2016) or ST or non-ST elevation myocardial infarction (Supreeth and Francis 2020). These cardiac conditions are associated with larger areas of fibrotic tissue (Scalise et al. 2021). As a consequence, a conduction delay in different regions of the ventricles is expected. However, myxomatous mitral valve disease consists of primary valve structural changes which induce hemodynamic dysfunction and myocardial wall stress (Richards et al. 2012). Nevertheless, several previous studies have shown that inflammation markers and fibrosis are also present in the myocardium of dogs with MMVD (Sakarin et al. 2016) especially in later stages (Falk et al. 2006), which may explain the high prevalence of nQRS. In humans with mitral valve prolapse, the average percentage of fibrosis infiltration is reported to be 28%, with a prevalence ranging from 13% in mild up to 37% in severe MR (Constant Dit Beaufils et al. 2021). Similar to our findings, one study regarding the diagnostic value of fQRS in people with heart failure with preserved ejection fraction (HFpEF) did not find any difference in the proportion of patients with fQRS among those who developed HFpEF and those who remained asymptomatic suggesting that fragmented QRS does not predict the development of heart failure symptoms (Karagodin et al. 2016).

These findings in relation to our results may support the idea the fibrosis infiltration in dogs with MMVD from one class to another is not as significant as to exhibit differences in nQRS and fQRS. In a previous study, most of the dogs with MMVD had one or two leads with a notched QRS. Moreover, when MMVD dogs were compared to dogs diagnosed with LV systolic dysfunction (considered to have dilated cardiomyopathy), the latter group tended to have more than two leads with a nQRS (Winter and Bates 2018). Notably, in humans with dilated cardiomyopathy, the presence of fQRS had been found to be associated with ventricular dyssynchrony (Supreeth and Francis 2020) which may also result in QRS notching.

This study has some limitations. Only standard six-lead electrocardiographic recording was available for analysis. A 12-lead ECG, including precordial leads may have offered additional information regarding the prevalence of notched QRS or the localization of fQRS regions in the left ventricle. Another limitation is that due to the retrospective design of the study, control group was chosen to be similar in age and BW to the MMVD group and no matched allocation was performed. This may have slightly influenced the results. However, the difference in prevalence of nQRS and fQRS between the two groups was clearly demonstrated.

## 5. Conclusions

This study did not identify any relationship between the number of leads with a notched QRS or fragmented QRS and disease severity nor with the age of dogs, however dogs with MMVD had a higher prevalence of both nQRS and fQRS compared to healthy dogs. Future prospective studies regarding the presence of notched QRS in 12 leads with evaluation of myocardial fibrosis by histopathology may be useful to correlate the electrocardiographic findings with the morphological changes of the myocardium.
